# Gallbladder mucocoele: A review

**DOI:** 10.4102/jsava.v86i1.1318

**Published:** 2015-12-09

**Authors:** Tesh M. Smalle, Alane K. Cahalane, Liza S. Köster

**Affiliations:** 1 Department of Companion Animal Clinical Studies, University of Pretoria, South Africa; 2 Veterinary Specialty Hospital of Hong Kong, Wan Chai, Hong Kong; 3 Department of Clinical Sciences and Center for Integrative Mammalian Research, Ross University School of Veterinary Medicine, West Indies

## Abstract

Gallbladder mucocoele (GBM) is an abnormal, intraluminal accumulation of inspissated bile and/or mucous within the gallbladder. Older, small- to medium-breed dogs seem to be predisposed, but no sex predilection has been identified. Clinical signs are often non-specific and include vomiting, lethargy, anorexia, abdominal pain, icterus and polyuria–polydipsia. Results of a complete blood count may be unremarkable, but serum biochemistry usually reveals increased liver enzymes. The ultrasonographic appearance is diagnostic and well described in the literature. Surgical intervention for the treatment of GBM remains the therapeutic gold standard, with short- and long-term survival for biliary surgery being 66%. The worst outcome is seen in those dogs requiring cholecystoenterostomy. With GBM becoming an apparently increasingly common cause of extrahepatic biliary disease in canines, it is essential that clinicians become familiar with the current literature pertaining to this condition. Numerous predisposing factors are highlighted in this review article and the role of certain endocrinopathies (e.g. hyperadrenocorticism and hypothyroidism) in the development of GBM is touched upon. Furthermore, the aetiopathogenesis of this disease is discussed with reference to the latest literature. Cholecystectomy remains the treatment of choice, but other options are considered based on a current literature review.

## Introduction

Over the past decade, gallbladder mucocoeles (GBM) have become a frequently recognised cause of extrahepatic biliary (EHB) disease in canines (Aguirre *et al*. 2007; Quinn & Cook 2009). This condition is rarely diagnosed in cats, with only two cases reported in the recent literature (Bennett *et al*. 2007; Woods *et al*. 2012).

Gallbladder mucocoeles are defined as an abnormal accumulation of inspissated, semi-solid bile and/or mucous within the gallbladder lumen (Mesich *et al*. 2009; Norwich 2011). The result is macroscopic distension of this organ with green-black gelatinous material that may extend throughout the biliary tree, causing variable degrees of EHB obstruction (Center 2009; Mesich *et al*. 2009). When EHB obstruction develops, gallbladder distension often leads to necrosis of the gallbladder wall and eventual rupture, with subsequent peritonitis (Worley, Hottinger & Lawrence 2004).

Its distinctive ultrasonographic appearance combined with historical, physical examination and serum biochemistry findings makes its diagnosis fairly easy (Center 2009; Kook *et al*. 2012). At the time of diagnosis, dogs are frequently suffering from concurrent problems that include pancreatitis, hyperlipidaemia, corticosteroid excess, hypothyroidism, protein-losing nephropathy, diabetes mellitus, cholestasis and gallbladder dysmotility (Aguirre *et al*. 2007). The mainstay of treatment is surgery, but medical management of selected cases has also been described (Quinn & Cook 2009).

Owing to its typical presentation, it is unlikely that the condition was previously misdiagnosed and its increasing incidence makes this an emerging syndrome in veterinary medicine.

## Anatomy

The gallbladder is a pear-shaped organ located in the right cranial abdominal quadrant. It is situated in the gallbladder fossa of the liver between the quadrate and right medial liver lobes. It consists of a fundus, body and neck that attaches, via a short cystic duct, to the common bile duct (Center 2009; Quinn & Cook 2009). The gallbladder wall consists of five histologically distinct layers. From the innermost these include the epithelium, submucosa (consisting of the lamina propria and tunica submucosa), tunica muscularis externa, tunica serosa (outermost layer covering the aspect of the gallbladder facing away from the liver) and tunica adventitia (outermost layer covering the aspect of the gallbladder facing towards the liver) (Quinn & Cook 2009). Its arterial supply is solely by the cystic artery (derived from the left branch of the hepatic artery), making this organ susceptible to ischaemic necrosis should its vascular supply become compromised (Center 2009). Its main function is as a storage reservoir for bile, where it can be concentrated (up to tenfold), acidified (through epithelial acid secretions) and modified (by the addition of mucin and immunoglobulins) before being released into the gastrointestinal tract at the major duodenal papilla (Aguirre *et al*. 2007; Center 2009; Quinn & Cook 2009). These functions, however, are not essential and cholecystectomy is well tolerated in numerous species, including dogs (Center 2009).

## Predisposing factors and aetiopathogenesis

The aetiology of GBM is incompletely understood, but is suspected to be complex and multifactorial (Norwich 2011).

Certain endocrinopathies (e.g. hyperadrenocorticism and hypothyroidism but not diabetes mellitus) may be implicated in the development of GBM (Meler & Pressler 2010). One study found that dogs previously diagnosed with hyperadrenocorticism were 29 times more likely to have findings of GBM, and comprised 21% of the GBM population in that study (Mesich *et al*. 2009). A separate study (*n* = 30) found 23% of dogs with GBM had hyperadrenocorticism (Pike *et al*. 2004).

In an attempt to create a GBM model, beagles received twice daily exogenous steroid administration (8.5 mg/kg hydrocortisone, q 12 hours) for 84 days to recreate hyperadrenocorticism (Kook *et al*. 2011, 2012). No significant differences were noted between treated dogs and controls, with sludge visible in the gallbladders of both groups. The most significant outcome of this experimental study was that the iatrogenic hypercortisolaemic state caused a reversible shift in bile salt composition towards an increased concentration of cytotoxic, hydrophobic, unconjugated bile acids. It has been demonstrated that these hydrophobic bile salts act as a mucin secretagogue in canine gallbladder epithelial cell cultures (Klinkspoor *et al*. 1995). From these experimental studies it can be postulated that alterations in bile acid cytotoxicity in the hypercortisolaemic states may be the cause of gallbladder dysfunction. Other hypotheses include an increased risk for the development of bacterial cholecystitis (as a result of concurrent immunosuppression) and alterations in gall bladder motility that may predispose to the development of GBM (Mesich *et al*. 2009).

Hypothyroid dogs were three times more likely to have GBM compared to euthyroid animals, but an observational bias may have resulted from dogs with confirmed GBM having an increased likelihood of being tested for hypothyroidism than control animals (Mesich *et al*. 2009). Thyroxine allows relaxation of the sphincter of Oddi, at the major duodenal papilla, of humans and pigs (Laukkarinen *et al*. 2002). Likewise, if it is deficient or absent, it is thought to cause increased tonicity of the sphincter and resultant biliary stasis. The increased contact time allows concentration of bile, leading to irritation of the gallbladder wall and increased mucous production. Thyroxine also affects bile acid composition, which may play a role in the development of GBM in hypothyroid dogs (Meler & Pressler 2010; Mesich *et al*. 2009).

Dyslipidaemias (due to hypothyroidism, hyperadrenocorticism and idiopathic hyperlipidaemia) seem to be associated with the development of GBM. This may be due to an increased conversion of cholesterol into bile acids as part of a catabolic escape pathway (Kook *et al*. 2012).

Interestingly, an insertion mutation in exon 12 of canine adenosine triphospate-binding cassette (ABCB4) may be a relative risk factor for the development of GBM in Shetland sheepdogs (Cullen 2009; Mealey *et al*. 2010). This mutation eliminates more than 50% of the functional protein. ABCB4 functions as a phospholipid translocator on the canalicular membrane of hepatocytes. In its absence, the concentration of phospholipids in the biliary lumen decreases, which increases the cytotoxicity of bile salts. The mutation is believed to be inherited in a dominant fashion with incomplete penetrance (Mahaffey 2011; Mealey *et al*. 2010).

Other steroid hormones (e.g. progesterone) result in concentration-dependent inhibition of gall bladder motility. Cholestasis may predispose animals to the development of GBM by increasing the contact time and allowing bile to become concentrated. This results in the irritation of the gallbladder wall and increased mucous production (Mesich *et al*. 2009; Quinn & Cook 2009).

Finally, the significance of biliary sludge in dogs and its association with the development of GBM remains unclear, but it is unlikely that the disease develops from normal bile without intermediate microprecipitate formation (Kook *et al*. 2012; Quinn & Cook 2009; Tsukagoshi *et al*. 2012).

Based on the current literature alone, it is apparent that many predisposing factors have been identified, but the aetiopathogenesis remains speculative in this species.

## Diagnosis

A summary of the signalment, clinicopathologic changes and bacteriology associated with gallbladder mucocoeles in dogs and cats is provided in Table 1.

**TABLE 1:** A summary of the signalment, clinicopathologic changes and bacteriology associated with gallbladder mucocoeles in dogs and cats generated by careful consideration of the mentioned reference list.

Description	Number	Percentage
**Number of animals reported in the literature**	-	-
Dogs	400	-
Total dogs where breed described	317	-
Cats	2	-
**Breeds**	-	-
Shelties	79	24.90
Cocker spaniels	56	17.60
Mixed breed	42	13.20
Miniature schnauzers	16	5.00
Bichon Frise	14	-
Miniature/toy poodles	12	-
Beagles	11	-
Dachshund	11	-
Pomeranians	9	-
Yorkshire terriers	7	-
Maltese poodle	6	-
Shih–tzu	6	-
German shepherd dog	6	-
Chihuahua	5	-
Labrador retriever	5	-
Min–pin	4	-
Collie breeds	4	-
Lhasa apso	3	-
Shiba inu	3	-
Basset hound	2	-
Border terrier	2	-
Cock-a-poo	2	-
Soft-coated wheaten terrier	2	-
Australian shepherd, Bernese mountain dog, Cairn terrier, golden retriever, Havanese, Jack Russell terrier, Keeshond, Norwich terrier, Pekingese, Siberian husky	1 of each	-
**Sex (where reported)**	332	-
Female	178	53.60
Male	154	46.40
**Mean age (years old)**	10.2	-
**Clinical signs (where reported)**	166	-
Vomiting	115	69.30
Lethargy	73	44.00
Anorexia	70	42.20
Abdominal pain/discomfort	32	19.30
Icterus	27	16.30
PU/PD	13	7.80
**Symptomatic vs. asymptomatic (where reported)**	122	-
Symptomatic	87	71.30
Asymptomatic	35	28.70
**Gallbladder (GB) rupture**	-	-
Cases where GB ruptured	45	27.80
Total number of GBM cases where GB rupture reported	162	-
**Clinicopathologic findings (where reported)**	-	-
Increased ALP	108/110	98.20
Increased ALT	90/103	87.40
Increased GGT	78/91	85.70
Increased total bilirubin	79/95	83.20
Increased AST	28/45	62.20
Increased cholesterol	15/27	55.60
Increased lipase	4/12	33.30
Increased amylase	13/44	29.50
Leucocytosis	23/49	46.90
**Bacterial culture**	-	-
Number of cultures performed	111	-
Number of positive cultures	15	13.50

GB, Gallbladder; GBM, Gallbladder mucocoele.

### Signalment

Older small- to medium-breed dogs seem to be predisposed (Norwich 2011). An average age of 9.1 years was found upon reviewing the current literature, but no sex predilection has been established (Aguirre *et al*. 2007; Besso *et al*. 2000; Choi *et al*. 2014; Crews *et al*. 2009; Malek *et al*. 2013; Mayhew, Mehler & Radhakrishnan 2008; Mealey *et al*. 2010; Mesich *et al*. 2009; Pike *et al*. 2004; Tsukagoshi *et al*. 2012; Uno *et al*. 2009; Walter *et al*. 2008; Worley *et al*. 2004). Shetland sheepdogs are predisposed to gallbladder disorders (comprising approximately 24.9% of reported cases), often with concurrent dyslipidaemia or gallbladder dysmotility (Aguirre *et al*. 2007; Crews *et al*. 2009). Cocker spaniels and miniature schnauzers are also over-represented (comprising 17.6% and 5% of reported cases, respectively) (Besso *et al*. 2000; Malek *et al*. 2013; Norwich 2011).

### Clinical signs

Clinical signs are often non-specific and include vomiting (approximately 69.3% of cases), lethargy (approximately 44.0% of cases), anorexia (approximately 42.2% of cases), abdominal pain (approximately 19.3% of cases), icterus (approximately 16.3% of cases) and polyuria-polydipsia (approximately 7.8% of cases) (Besso *et al*. 2000; Pike *et al*. 2004). The duration of signs is typically for 5 days prior to presentation, with only 71.3% of cases displaying clinical signs (Besso *et al*. 2000; Choi *et al*. 2014; Escobar & Neel 2011; Mayhew *et al*. 2008; Norwich 2011; Pike *et al*. 2004; Reed, Ramirez & American College of Veterinary Radiology 2007; Walter *et al*. 2008; Worley *et al*. 2004).

### Clinicopathologic findings

The results of a complete blood count are usually unremarkable with leukocytosis evident in 46.9% of cases. The leukocytosis can be characterised by a left shift neutrophilia (regenerative or degenerative) (Worley *et al*. 2004). Serum biochemistry reveals increased liver enzymes, including alkaline phosphatase (98.2% of cases), alanine aminotransferase (87.4% of cases), gamma-glutamyltransferase (85.7% of cases) and aspartate aminotransferase (62.2% of cases). Hyperbilirubinaemia is reported in 83.2% of the cases in the current literature (Center 2009; Malek *et al*. 2013). Amylase and lipase are also elevated in some cases: 29.5% and 33.3% of reported cases, where measured on serum biochemistry, respectively (Malek *et al*. 2013; Worley *et al*. 2004). Cholesterol is often elevated (55.6% of reported cases where measured) (Worley *et al*. 2004). However, the most significant biomarkers associated with outcome were found to be elevated mean serum lactate concentration and decreased packed cell volume (Malek *et al*. 2013).

### Ultrasonography

**FIGURE 1:**
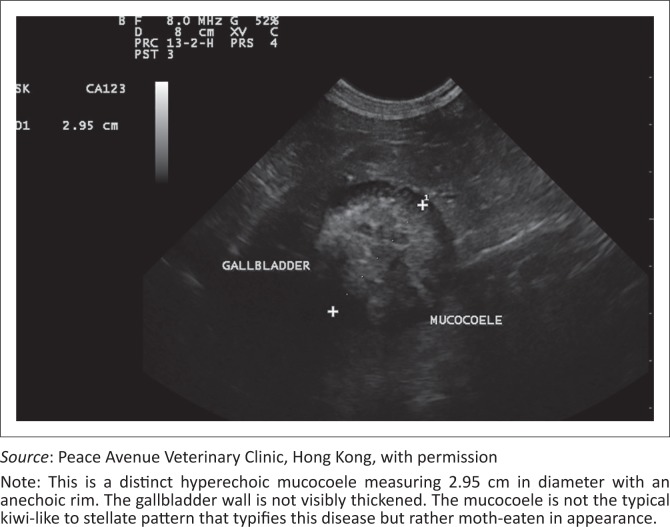
Ultrasonographic image (8 MHz convex array probe, Esaote, MyLab, Genoa, Italy) of a sagittal section of a gallbladder in a dog diagnosed with a gallbladder mucocoele.

**FIGURE 2:**
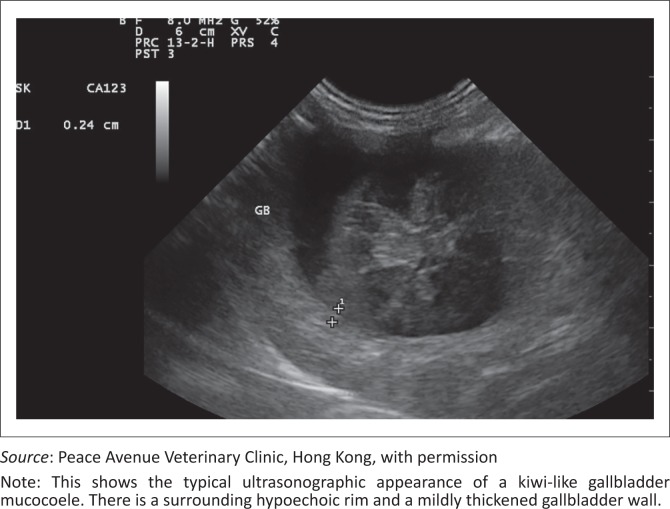
Ultrasonographic image (8 MHz convex array probe, Esaote, MyLab, Genoa, Italy) of a sagittal section of a gallbladder in a dog diagnosed with a gallbladder mucocoele.

The ultrasonographic appearance is diagnostic and well described in the literature. The classic GBM was described in earlier studies as a finely striated stellate pattern that differs from biliary sludge in that it is non-dependent (Besso *et al*. 2000). The most common abdominal ultrasonographic findings in dogs with clinically significant GBM include echogenic peritoneal fluid (more frequently confined to the gallbladder fossa), a thickened or laminated gallbladder wall, and an echogenic reaction in the gallbladder fossa (Crews *et al*. 2009). Ultrasonographic signs of gallbladder rupture were 100% specific and included discontinuity of the gallbladder wall, hyperechogenicity of the cranial abdominal fat, free peritoneal fluid, or a free, well-organised mucocoele within the peritoneal cavity (Center 2009; Crews *et al*. 2009; Gaschen 2009) (Figures 1 & 2).

A study of 11 dogs that underwent cholecystectomy for GBM examined the relationship between the morphological appearance of the gallbladder content and the ultrasonographic pattern (Uno *et al*. 2009). This study described three ultrasonographic patterns, namely: pattern one, hyperechoic content filling the entire gallbladder or precipitated immobile content; pattern two, a thin hypoechoic exterior layer separated by an indistinct border from an internal hyperechoic area containing moth-eaten to mosaic-form hypoechoic areas; and pattern three, a thick hypoechoic area in the exterior layer separated by a distinctive border from a prominent internal hyperechoic area. This study concluded that pattern two was a forerunner of the typical kiwi fruit pattern diagnostic for GBM and should indicate the gallbladder dysmotility and cholangiohepatitis that often accompany this disease.

A more recent study examined the ultrasonographic findings and attempted to correlate these with clinical signs (Choi *et al*. 2014). A larger group of dogs (*n* = 43) was divided into six groups based on the ultrasonographic pattern, namely: type 1, immobile echogenic bile (23%); type 2, incomplete stellate pattern (30%); type 3, typical stellate pattern (12%); type 4, kiwi-like pattern and stellate combination (26%); type 5, kiwi-like pattern with residual central echogenic bile (9%); and type 6, kiwi-like pattern (0%). The ultrasonographic pattern was not associated with the clinical signs and is thus not a valid diagnostic tool for grading disease.

Gallbladder motility can also be evaluated ultrasonographically. Tsukagoshi *et al*. (2012) developed a gallbladder ejection fraction index that could be used to estimate dysmotility. Gallbladder volume was estimated on a longitudinal image post-12 hour starve and then 60 and 120 min postprandial (Hills a/d at 10 g per kilogram body weight) in order to calculate this index. This study proposes that gallbladder dysmotility is important in the aetiopathogenesis of GBM due to the increased exposure time of epithelial cells to bile salts.

### Scintigraphy

Hepatobiliary scintigraphy is helpful in determining bile duct patency (Head & Daniel 2005). Findings in animals with chronic, complete EHB obstruction include reduced hepatic extraction fraction (which represents the portion of the radiopharmaceutical that is removed from the plasma on each circulatory pass through the liver), prolonged clearance half-life, inability to view the biliary tree and absence of radioactivity in the intestine (Head & Daniel 2005).

Hepatobiliary scintigraphy was performed in three dogs in the diagnostic work-up of GBM (Worley *et al*. 2004). Dogs were given 2–3 mCi of technetium Tc 99m-mebrofenin intravenously. All the scintigraphic scans revealed complete obstruction of the extrahepatic biliary system. This diagnostic technique requires registered facilities and has a considerable health and safety risk with accuracies that do not compete favourably with ultrasound.

### Bacteriology

Approximately 13.5% of GBM have concurrent bacterial colonisation of the gallbladder (Amsellem *et al*. 2006; Malek *et al*. 2013; Mayhew *et al*. 2008; Pike *et al*. 2004; Uno *et al*. 2009; Worley *et al*. 2004). Ultrasound-guided cholecystocentesis is advised to collect a bile sample for bacterial culture and antibiogram testing in animals where medical management is pursued for cholecystitis. However, ultrasound-guided cholecystocentesis should be performed with caution if GBM is suspected as complication rates have not been determined (Center 2009). Complications associated with this procedure include bile leakage, bradycardia due to vagal stimulation, bacteraemia and local haemorrhage (Quinn & Cook 2009).

### Histopathology

Cystic mucosal hyperplasia is often seen on microscopic examination of the affected gallbladder wall. Transmural ischaemic necrosis may be seen in the fundic region and precedes gallbladder rupture (Center 2009). The submission of all gallbladders post cholecystectomy is advised.

## Treatment

### Medical management

Large standardised prospective studies are still lacking for medical management of GBM and thus it cannot be recommended as the first line of treatment in dogs. It can be considered in asymptomatic animals and there are individual case reports supporting its use (Norwich 2011; Walter *et al*. 2008). However, all reported cases had concurrent hypothyroidism that needed to be addressed before resolution of GBM was possible.

Owners must be made aware that asymptomatic cases may develop into an acute clinical emergency should the disease progress to extrahepatic biliary obstruction or gallbladder rupture (Norwich 2011; Quinn & Cook 2009).

The foundation of medical management includes the use of choleretics and hepatoprotectants. Ursodeoxycholic acid is a naturally occurring hydrophilic bile acid that functions as a choleretic and hepatoprotectant at 10 mg/kg – 15 mg/kg PO, as a single dose or divided into two doses per day. S-adenosylmethionine is a naturally occurring precursor of cysteine that is essential in the production of the antioxidant glutathione, and therefore is hepatoprotectant at 18 mg/kg – 20 mg/kg PO, administered once daily on an empty stomach (tablets must not be split). A low-fat diet is encouraged, especially in animals with dyslipidaemias, as dietary management may stimulate biliary flow (Mitchell 2010; Norwich 2011). Concurrent endocrinopathies must be treated appropriately.

Regular monitoring of medically managed cases is recommended, with follow-up visits every 2–4 weeks for abdominal ultrasound, haematology and serum biochemistry. Any progression of the disease process warrants immediate surgical intervention (Center 2009; Quinn & Cook 2009).

### Surgery

Surgical intervention for the treatment of GBM remains the therapeutic gold standard. Pre-operative haematology, serum biochemistry, urinalysis and coagulation profiles are recommended in all cases. Supplementing animals with vitamin K_1_ has been advocated pre-operatively, even in the light of normal coagulation profiles. This can be instituted by administering three doses of vitamin K_1_ at 0.5 mg/kg SC every 12 hours prior to surgery (Mitchell 2010; Quinn & Cook 2009). However, there are no published studies that support this recommendation.

Cholecystectomy is the recommended surgical procedure for the treatment of GBM. Microscopic examination of the gallbladder wall in cases of GBM indicates that it is diseased and this warrants its removal. However, it is crucial that the patency of the common bile duct is confirmed prior to cholecystectomy, either via manual normograde expression of the gallbladder or retrograde catheterisation of the common bile duct via duodenal enterotomy. The biliary tree must be flushed prior to ligation of the cystic duct in order to remove residual thick, inspissated bile and/or small choleliths to minimise the risk of post-operative biliary obstruction. The resected gallbladder is submitted for microscopic examination and bacterial culture. Liver biopsy (for histopathology, copper, iron and zinc concentrations) is indicated in all cases.

Laproscopic cholecystectomy has been described for cases where biliary tract obstruction or rupture has been excluded (Mayhew *et al*. 2008).

Cholecystotomy is not recommended as microscopic mural necrosis may be present and could result in post-operative gallbladder rupture. There are also documented cases of recurrence of GBM formation in dogs where cholecystotomy was initially performed (Center 2009).

Cholecystoenterostomy is feasible for rare GBM cases in which the patency of the common bile duct cannot be established. This procedure is, however, associated with a higher complication rate.

Recommended peri-operative care includes feeding a low-fat diet and broad-spectrum antimicrobial and hepatoprotectant therapy as described for medical management (Quinn & Cook 2009).

## Prognosis and conclusion

The short- and long-term survival for biliary surgery is 66%, with the worst outcome in those dogs requiring cholecystoenterostomy (Amsellem *et al*. 2006). A peri-operative mortality rate of 21.7% – 40% is reported for dogs undergoing cholecystectomy for GBM. Most mortalities occur within the first 2 weeks after surgery, with long-term survival beyond this point being excellent. The most common complications include bile peritonitis, sepsis, disseminated intravascular coagulation and surgical-site dehiscence (Norwich 2011). Dogs diagnosed with concurrent pancreatitis are considered to have a poor prognosis (Amsellem *et al*. 2006). Elevation of the serum lactate concentration post-operatively and post-operative hypotension were significantly associated with a poorer clinical outcome (Malek *et al*. 2013). Conversely, published evidence indicates that the survival rate is not affected by bile leakage from gallbladder rupture nor from concurrent bacterial colonisation of the bile (Crews *et al*. 2009; Pike *et al*. 2004).

A small study examining 11 cases of GBM treated surgically and their corresponding ultrasonographic pattern found significant differences between the survivors and the non-survivors (Uno *et al*. 2009). The dogs that died were older (mean age 11.8 ± 1.5 years) and had higher white cell counts (46 600 ± 11 912/μL) than the survivors, which had a mean age of 8.4 ± 2.8 years and a white cell count of 18 266 ± 9411/μL.

With GBM becoming an apparently increasingly common cause of extrahepatic biliary disease in canines, it is essential that clinicians become familiar with the current literature pertaining to this condition. However, further prospective studies with larger case numbers are necessary to clarify the intricacies of this disease.
